# Supplementary material for: The Cascade of Care for Hepatitis C Treatment in Rwanda: A Retrospective Cohort Study of the 2017–2019 Mass Screening and Treatment Campaign
Source: Viruses. 2023 Feb 28;15(3):661. doi: 10.3390/v15030661 (PMC10056983; doi:10.3390/v15030661)
Supplement: Supplementary file 1 [file viruses-15-00661-s001.zip › viruses-2140175-supplementary.pdf]

Table S1. Patients' characteristics per treatment outcome.

Variables	Total sample	Cured	Failed	Death	Discontinuation	Total
<44	1,591	13	14.4	0	10.2	12.3
44-54	1,573	13.5	14	3.6	8.7	12.2
55-64	2,977	25.1	24.9	16.1	17.6	23
64+	6,799	48.4	46.7	80.4	63.5	52.5
Total	12,940	100	100	100	100	100
Sex						
Female	7,965	60.8	59.3	48.2	64.3	61.6
Male	4,974	39.2	40.7	51.8	35.7	38.4
Total	12,939	100	100	100	100	100
Social Economic Status						
Category 1	2,587	23.9	26.6	29.2	31	25.8
Category 2	3,305	33.7	32.1	18.8	31.7	33
Category 3 or 4 or unknown	4,124	42.5	41.3	52.1	37.2	41.2
Total	10,016	100	100	100	100	100
Marital status						
Single	3,780	12.1	25.1	25	70.3	29.2
Married or in union	6,390	61.6	52.3	42.9	20.3	49.4
Separated/divorced/Widow	2,770	26.3	22.6	32.1	9.4	21.4
Total	12,940	100	100	100	100	100
Health Insurance						
No insurance	2,611	4.7	10.5	16.1	59	20.2
Community insurance	9,942	91.8	86.4	75	39.2	76.8
Private or other government insurance RAMA/RSSB)	387	3.5	3.1	8.9	1.8	3
Total	12,940	100	100	100	100	100
Diabetes status						
No	9,688	97.3	96.5	84.4	97.9	97.2
Yes	274	2.7	3.5	15.6	2.1	2.8
Total	9,962	100	100	100	100	100
HIV status						
No	8,970	89.3	90.5	77.3	94.2	90
Yes	1,002	10.7	9.5	22.7	5.8	10
Total	9,972	100	100	100	100	100
Cancer status						
No	9,827	98.7	99.1	86.7	99.3	98.7
Yes	126	1.3	0.9	13.3	0.7	1.3
Total	9,953	100	100	100	100	100
Family History of Viral Hepatitis						
No	9,253	95.1	92.9	95.5	98.3	95.3
Yes	459	4.9	7.1	4.5	1.7	4.7
Total	9,712	100	100	100	100	100
Renal failure status						
No	12,769	98.2	99	96.4	99.7	98.7
Yes	171	1.8	1	3.6	0.3	1.3
Total	12,940	100	100	100	100	100
HTA status						
No	11,672	87.5	88.5	76.8	97.3	90.2
Yes	1,268	12.5	11.5	23.2	2.7	9.8
Total	12,940	100	100	100	100	100
HBV result						
Negative	10,421	98.6	97.6	100	98.5	98.5
Positive	163	1.4	2.4	0	1.5	1.5
Total	10,584	100	100	100	100	100
HCV treatment history						
No	11,701	88.8	85.1	96.4	99.7	91.6
Yes	1,077	11.2	14.9	3.6	0.3	8.4
Total	12,778	100	100	100	100	100
Treatment regimen						
HARVONI	3,452	37.2	22	42.9	3.6	26.7
SOF+DCV	4,761	52.7	31.5	17.9	1.8	36.8
Other combination	4,727	10.1	46.5	39.3	94.7	36.5

Total	12,940	100	100	100	100	100
Ever been traditionally operated						
No	8,009	81.2	78.5	66.7	84.3	81.3
Yes	1,848	18.8	21.5	33.3	15.7	18.7
Total	9,857	100	100	100	100	100
Ever been transfused						
No	9,584	96.8	96.8	91.1	98.5	97
Yes	297	3.2	3.2	8.9	1.5	3
Total	9,881	100	100	100	100	100
Ever been operated						
No	9,552	96.5	96	93.3	96.3	96.4
Yes	353	3.5	4	6.7	3.7	3.6
Total	9,905	100	100	100	100	100
Mean baseline Viral load(IU/mL)	12,940	2,092,958	3,712,036	1,030,728	1,948,949	2,155,255
Mean number of hospital staff	12,940	224	214	363	256	232

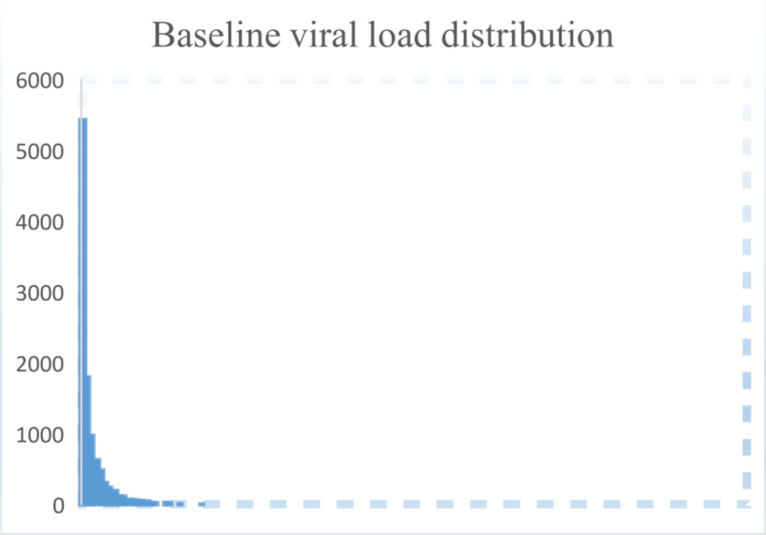

Figure S1. Baseline viral load distribution plot.
